# Herbal Supplement-Induced Liver Injury: A Case Report

**DOI:** 10.7759/cureus.33663

**Published:** 2023-01-11

**Authors:** Haidar Khan, Jonathan Vincent M Reyes, Tasur Seen, Branden Irefej, Saad Ahmad

**Affiliations:** 1 Internal Medicine, Icahn School of Medicine at Mount Sinai, Elmhurst Hospital Center, Elmhurst, USA; 2 Internal Medicine, Icahn School of Medicine at Mount Sinai, Elmhurst Hospital, Elmhurst, USA

**Keywords:** herbal-induced liver injury (hili), liver damage, herbal medications, drug-induced liver injury (dili), hepatitis, herbal supplements

## Abstract

We present the case of a 45-year-old woman who arrived at the emergency department complaining of sudden epigastric pain. An inpatient evaluation revealed no evidence of viral or immunologic infection. Additionally, imaging did not elicit a clear cause for the patient’s symptoms. Further examination revealed that the patient had recently begun using a herbal tea and that symptoms had completely resolved after discontinuation. Though rare, hepatotoxicity secondary to herbal supplement ingestion, or herbal supplement-induced liver injury, or HILI, should be considered in all patients presenting with abnormal liver function tests.

## Introduction

Herbal supplements have seen an increase in acceptance among Western countries recently as an alternative to prescribed medication. Due to a lack of regulations regarding their manufacturing and marketing, supplements are often erroneously perceived by the public as having little to no side effects. Though rare, this misconception has led to herbal supplement-induced liver injury (HILI) in susceptible individuals. HILI is a rare but often overlooked cause of liver disease. It should be taken into consideration after an extensive workup fails to produce a definitive diagnosis for an unspecified liver injury.

## Case presentation

A 45-year-old woman presented with one day of severe epigastric pain and nausea. She had a past medical history of hypothyroidism, and her only medication was levothyroxine. The patient denied drug or alcohol use, recent travel, and a history of blood transfusions. Of note, she began drinking herbal tea daily for three days before experiencing symptoms to "improve immunity." The exam was notable for a non-tender abdomen and a lack of jaundice and organomegaly. Laboratory values were significant for elevated bilirubin (total 1.9 mg/dL and direct 1.2 mg/dL), alkaline phosphatase (145 IU/L), aspartate aminotransferase (1463 IU/L), alanine transaminase (940 IU/L), gamma-glutamyl transpeptidase (215 IU/L), and lactate dehydrogenase (818 IU/L). The Hepatitis B surface antibody was reactive, but the hepatitis B surface antigen, E antigen, core antibody, hepatitis C antibody, and hepatitis A antibodies were non-reactive. Ceruloplasmin, antinuclear antibody, anti-smooth muscle antibody, and iron panel were within normal limits. Ultrasound of the right upper quadrant was significant for stone and sludge with a 5 mm gallbladder wall. Magnetic resonance cholangiopancreatography (MRCP) revealed cholelithiasis without cholecystitis. The patient was observed without further intervention, and transaminases and bilirubin started decreasing on the third day of admission and decreased until discharge on the fifth day. Further investigation revealed 23 different ingredients in the herbal tea, most notably reishi mushroom (Ganoderma lucidum), aloe vera (Aloe barbadensis), and Siberian ginseng (Eleutherococcus senticosus). She did not drink the tea during her hospital stay and was advised to discontinue use after discharge. By the three-month follow-up, the patient’s bilirubin, transaminases, and alkaline phosphatase had all normalized.

## Discussion

It is estimated that roughly one-third of Americans take herbal supplements [1]. Due to their categorization as supplements, they are not as closely regulated as traditional pharmaceuticals, despite heavy marketing and various purported health claims. As a result, formal studies examining the efficacy and side effects of various supplements are limited by the unreliability of ingredient labeling, dosing, and possible contamination. Aloe vera is a "likely but rare cause of clinically apparent liver injury," with about 12 case reports published since 2005 [2]. Yuen MF et al. and Wanmuang HT et al. identified acute liver injuries in otherwise healthy patients who began using reishi mushrooms [3,4]. Lastly, Enioutina EY et al. examined reports of patients concurrently using Siberian ginseng along with other hepatically cleared prescribed medications and developing acute liver injury [5]. The article suggests ginseng may inhibit various cytochromes, though the data remain equivocal.

## Conclusions

A diagnosis of HILI requires an effective inquiry into any herbs or supplements a patient may be taking, as patients may be hesitant to provide this information. This case shows the value of further inquiry into supplement use once common causes of acute liver injury are ruled out. In this case, the patient was using an herbal tea with known hepatotoxic ingredients and had definitive improvement after cessation of use. It is imperative for clinicians to familiarize themselves with herbal supplements to better inquire about their use with patients and educate them on potential side effects.
